# Dyadic Internalized Stigma, Resilience, and Psychological Distress Among People with HIV and their Family Members: An Actor-Partner Interdependence Mediation Model Analysis

**DOI:** 10.1007/s10461-026-05104-7

**Published:** 2026-03-26

**Authors:** Fanghui Shi, Xueying Yang, Shan Qiao, Cheuk Chi Tam, Shuaifeng Liu, Yuejiao Zhou, Xiaoming Li

**Affiliations:** 1 South Carolina SmartState Center for Healthcare Quality, University of South Carolina Arnold School of Public Health, Columbia, SC 29208, USA; 2 Department of Health Promotion, Education and Behavior, Arnold School of Public Health, University of South Carolina, Columbia, SC 29208, USA; 3 Guangxi Center for Disease Control and Prevention, Nanning, China

**Keywords:** People with HIV, Family member, Internalized stigma, Resilience, Psychological distress

## Abstract

Despite robust evidence showing the mediation role of psychological resilience in the impacts of HIV-related stigma on psychological distress, most studies were conducted from an intrapersonal perspective. Limited studies have examined the dyadic effects of HIV-related stigma and resilience on psychological distress for people with HIV (PWH) and their family members. This study aimed to explore the actor-partner effect and the mediation role of resilience in the relationship between internalized stigma and psychological distress among PWH and family members dyads. Between October 2023 and December 2023, a total of 800 PWH-family member dyads were recruited from two urban cities and nine rural counties in Guangxi, China. All participants completed validated questionnaires capturing demographic characteristics, internalized stigma, resilience, and psychological distress. This study employed the actor-partner interdependence mediation model to examine both individual (actor) and dyadic (partner) pathways, along with resilience as a mediator. For actor effects, resilience partially mediated the relationship between internalized stigma and psychological distress in PWH (β = 0.08, SE = 0.01, *p* < 0.001) but not in their family members (β = −0.00, SE = 0.01, *p* = 0.68). Regarding partner effects, indirect effects were significant in the association between family members’ internalized stigma and PWH’s psychological distress through PWH’s resilience (β = 0.03, SE = 0.01, *p* < 0.01). This study underscores the complex interplay between internalized stigma, resilience, and psychological distress in PWH-family member dyads. Psychological health promotion programs would benefit from adopting family-centered strategies that attend to stigma reduction and resilience improvement for both PWH and family members.

## Introduction

Due to detrimental impacts on HIV care and prevention (e.g., poor medication adherence and increased HIV-risk behaviors), psychological distress (e.g., depression and anxiety) is among the most critical challenges for people with HIV (PWH), and it has been of particular concern in the Chinese context [1, 2]. Recent systematic review studies have consistently revealed the high prevalence of psychological distress in PWH (44% overall; 31% depression; 29% anxiety), and such rates are higher in Asian regions (e.g., 38% depression) and developing countries (48% overall), including China [3, 4]. Their family members (either immediate family or family of choice) [5–8], who typically play a long-term role in providing care and support, have also experienced high vulnerability to psychological distress [9, 10]. For example, studies from China have demonstrated that psychological distress is common in family members (17%), with very high rates of distress-related experiences (e.g., 85% negative feelings and 54% poor quality of life) [11, 12].

A substantial body of research has investigated factors related to psychological distress, and HIV-related stigma (e.g., enacted, perceived, and internalized stigma) has consistently been identified as a significant risk factor [13–15]. Internalized HIV stigma, in particular, refers to the endorsement of negative beliefs, views, and feelings of oneself due to one’s HIV-positive status [15, 16], and it may be shaped by broader social experiences, such as exposure to prejudice and discrimination (i.e., enacted stigma). Internalized stigma has been recognized as a proximal psychological mechanism that may influence psychological outcomes among PWH [17, 18]. Notably, family members are also susceptible to HIV-related stigma. This phenomenon has been described as courtesy stigma, which refers to the stigma experienced by both the stigmatized person and his/her intimates who are considered as one unit in a social environment, even though they might not be infected by HIV [19, 20]. Courtesy stigma may manifest internally as shame or self-blame, especially among family members who provide care and emotional support for PWH [21, 22].

Interdependence Theory offers a framework to understand that individuals in close relationships can change their feelings, thoughts, and behavior in response to one another’s needs and goals, resulting in shared outcomes at the dyadic level [23]. In the context of HIV stigma, PWH’s psychological distress may not only stem from their own stigma experience (i.e., actor effect) but also be shaped by their family members’ stigma experience (i.e., partner effect), and vice versa, as each partner adapts their emotional and behavioral responses in light of their relational dynamics. Given the emphasis on family values in Chinese culture, such a dyadic association could be particularly pronounced within Chinese families of PWH. A handful of Chinese studies have examined dyadic effects of HIV-related stigma among PWH and close others (e.g., caregivers and couples) and indicated significant effects on various health outcomes (e.g., quality of life and dignity-related distress) [24, 25].

The dyadic association of internalized stigma with psychological distress may be mediated by intrapersonal coping resources, such as resilience. Resilience refers to one’s personal resources and ability to cope with or overcome adversities and stress, while its manifestation would be influenced by HIV-related stigmatization experiences [26]. The burden of self-devaluation and negative self-perception associated with internalized stigma could impair an individual’s sense of self-efficacy, optimism, and mastery, which are essential components of resilience [27, 28], and these influences could extend to psychological distress. Existing Chinese HIV-related stigma research has demonstrated the mediation model of resilience, in which higher HIV-related stigma is associated with higher psychological distress indirectly through resilience [29–32]. A study involving 2,987 Chinese PWH found that individual-level resilience resources mediated the association between internalized stigma and self-reported mental health status [33]. Notably, beyond the individual aspect, this mediation role of resilience may also operate at the dyadic level with families of PWH. Such a dyadic effect of resilience on psychological health outcomes has been documented in Chinese family member studies of individuals with chronic conditions (e.g., cancers and spinal cord injury), fostering a perspective to examine the role of resilience within families of PWH from a dyadic level [34–36].

**Fig. 1:**
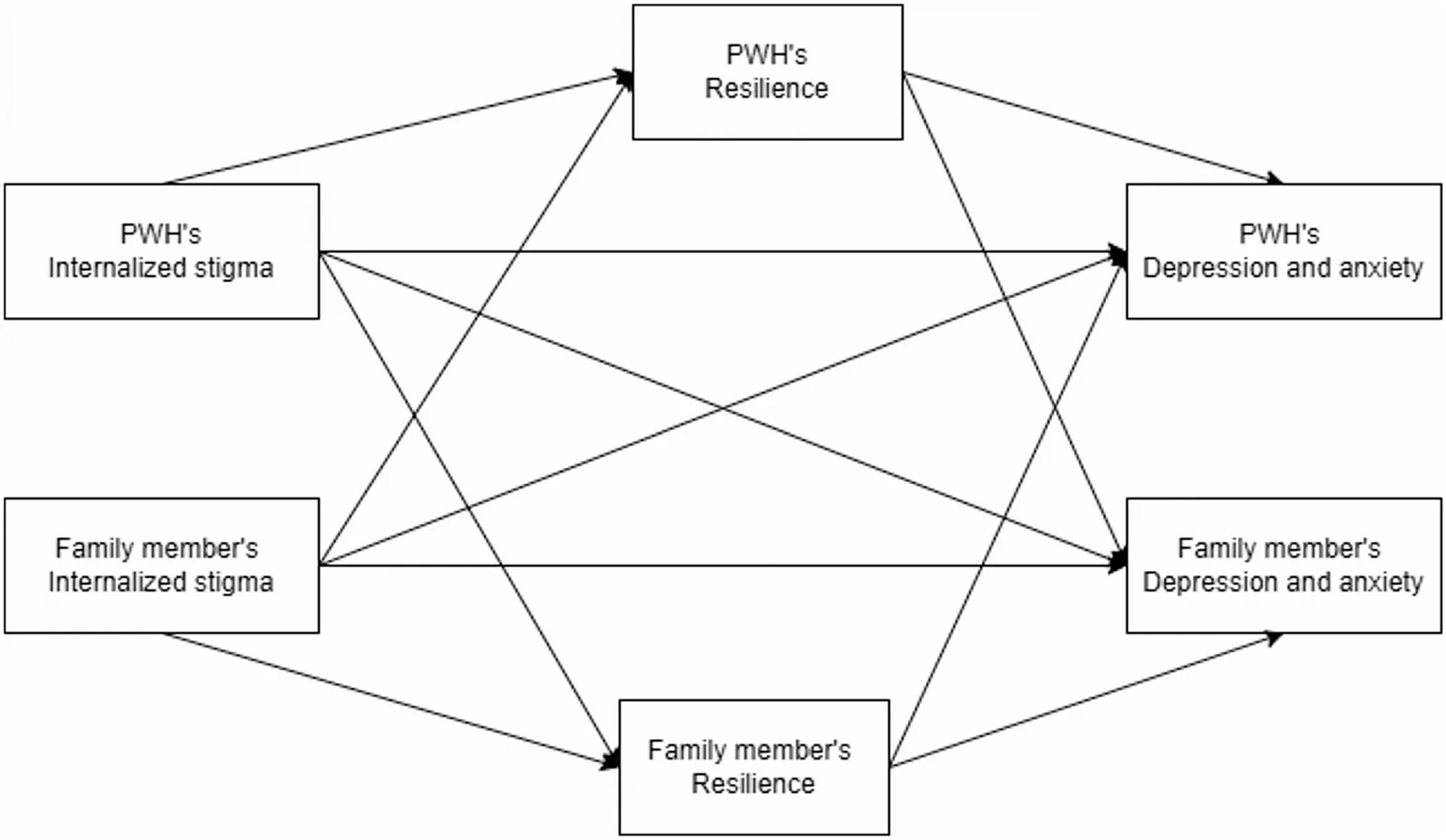
The hypothesized actor-partner interdependence mediation model

Despite the growing evidence showing the potential interdependence between PWH and their family members, understanding of the mechanisms through which internalized stigma may influence psychological distress across PWH-family member dyads, and the potential mediating role of resilience at both the actor and partner levels, remains limited. Guided by the Interdependence Theory and previous research on resilience [29–32], the present study aims to examine the actor-partner effect in the mediation among internalized stigma, resilience, and psychological distress in a dyadic relationship between Chinese PLW and their family members. In particular, we hypothesized that (1) internalized stigma in PWH and their family members would be positively associated with their own and family members’ psychological distress; (2) the dyadic association between internalized stigma and psychological distress would be mediated by resilience from PWH and family members (see Fig. 1). The examination of this hypothesized model could extend the literature by providing novel insight into how internalized stigma-related distress is jointly experienced and interconnected within family dyads. The findings have the potential to inform the development of dyad-based, resilience-focused interventions aimed at mitigating the psychological burden of internalized stigma among PWH and their family members.

## Methods

### Participants and Procedures

Data for this study were drawn from the baseline assessment of an ongoing intervention study aiming to mitigate the effect of HIV-related stigma through resilience-based intervention among PWH and their key supporting systems (e.g., family members and healthcare providers) in China [37]. In the current study, dyadic data from PWH and their family members were extracted for analyses. From October 2023 to December 2023, participants were recruited from two cities and nine rural counties that have the largest number of reported HIV cases in Guangxi. Guangxi is one of the regions that is experiencing the fastest growth of the HIV epidemic in China, with more than 70,000 HIV cases reported by the end of 2022 [38].

The eligibility criteria for PWH for the present study included the following: (a) aged 18 years or older; (b) diagnosed with HIV in the past two years, detectable viral load (e.g., viral load above 50 copies/mL), or a viral rebound during the past year (i.e., detectable viral load following a suppression); (c) willing to refer one of the adult family members (either of origin or of choice) to participate; (d) willing to provide a hair sample for testing hair cortisol and antiretroviral concentration; (e) willing to consent for the retrieval of viral load and CD4 count data from their medical records; (f) willing to receive intervention at different timepoints in the stepped wedge trial. For family members, the inclusion criteria include: (a) aged 18 years or older; (b) referred by PWH enrolled in the study; (c) either an immediate family member (i.e., parents, siblings, spouses, and children) or family of choice (i.e., individuals who play a supportive family role but not fall into the immediate family category, such as close friends and extended family members); and (d) willing to be randomly assigned intervention along with PWH. A total of 800 PWH and 800 family members enrolled in the study.

In Guangxi, there is one designated primary public hospital with an HIV clinic in each urban district and rural county that works under the direction of the Centers for Disease Control and Prevention (CDC) to conduct case management for all PWH in each urban district or rural county. In the two cities and nine rural counties that have the largest number of reported HIV cases in Guangxi, a systematic random sampling procedure was used to randomly select prospective participants from the HIV patient registries system of the public HIV clinics in each city or county [37]. Eligible participants and their family members were contacted and provided with an informed consent form outlining the study purposes, confidentiality, and voluntary nature. After obtaining consent, participants were invited to complete a baseline 45-minute interviewer-administered questionnaire in private rooms at the local CDC offices. The interview provided explanations and clarification for the questions upon participants’ request. Upon completion, participants received RMB 100 (equivalent to 13.73 USD) as an incentive.

### Measures

#### Sociodemographic and Clinical Characteristics

To determine sociodemographic background of PWH and their family members, the following categorical information was collected: age, sex (i.e., male or female), living in rural areas (i.e., yes or no), education attainment (i.e., less than primary school, primary school, middle school, and high school or higher), employment status (i.e., employed, unemployed, and other), and perceived household income level (i.e., lower than average, average, and above average). For PWH, we collected information regarding HIV transmission mode (i.e., sex, blood donation, injection drug use, and other/unknown). For family members, we collected information about their HIV status (i.e., positive, negative, and unknown) and family relationship type (i.e., immediate family and family of choice).

#### HIV-related Internalized Stigma

For PWH, HIV-related internalized stigma evaluated personal feelings and self-evaluations associated with living with HIV and was assessed using six items from the Negative Self-Image Scale, a subscale of the HIV Stigma Scale [39]. Sample items included statements such as “*I feel I’m not as good as others because I have HIV*” and “*Having HIV makes me feel unclean*.” Response choices ranged from 1 = strongly disagree to 5 = strongly agree.

For family members, a similar measure was employed to assess HIV-related courtesy internalized stigma, with six items adapted from the Negative Self-Image subscale of the HIV Stigma Scale [20]. These items were modified to reflect the experience of living with or supporting someone with HIV, rather than being with HIV by themselves [20]. Example items include, “*Having a family member with HIV makes me feel I am worse than others*,” and “*Having a family member with HIV makes me feel ashamed*.” A total courtesy internalized stigma score was computed by summing responses to all items, with higher scores indicating greater courtesy internalized stigma. In this study, we conceptualize internalized stigma among family members as reflecting one dimension of courtesy stigma, the internal experience of stigma resulting from association with a stigmatized individual. The internal consistency of the internalized stigma scale was excellent in this study, with the Cronbach’s alphas being 0.97 for PWH and 0.91 for their family members.

#### Resilience

Resilience for PLW and family members was measured using the 10-item Conner-Davidson Resilience Scale (CD-RISD), which assesses positive adaptation to stress or trauma [40, 41]. Sample items included “*Able to adapt to change*,” “*Can stay focused under pressure*,” and “*Can deal with whatever comes*.” Responses were rated on a 5-point scale ranging from 1 = never to 5 = always. A total score was computed by summing responses to all items, with higher scores indicating stronger resilience. The scale exhibited good internal reliability in the current study (Cronbach’s alpha = 0.97 for PWH and 0.96 for their family members).

#### Psychological Distress

Psychological distress was assessed with the Patient Health Questionnaire for Depression and Anxiety (PHQ-4), a four-item scale that included two items measuring depression and two items measuring anxiety [42]. The PHQ-4 began with the stem questions: “Over the last two weeks, how often have you been bothered by the following problems?” The problems listed included “*No interest (or pleasure) in doing things*,” “*Feeling down*, *depressed or hopeless in life*,” “*Feeling nervous*, *anxious or uneasy*,” and “*Cannot stop or control worry*.” Responses were scored as 1 = rarely or none of the time (less than 1 day per week), 2 = some or a little of the time (1–2 days per week), 3 = occasionally or a moderate amount of time (3–4 days per week), and 4 = all of the time (5–7 days per week). Thus, the total score ranged from 4 to 16, with higher values indicating greater psychological distress. The Cronbach’s alphas of this scale in the current study were 0.94 and 0.95 for PWH and their family members, respectively.

### Statistical Analysis

First, data were screened in terms of missing values, outliers, and normality (for continuous variables). Descriptive statistical analyses, including frequency, percentage, Mean, and standard deviation (SD), were conducted to describe the demographic characteristics and study variables between PWH and family members. A paired t-test was conducted to examine the differences in internalized stigma, resilience, and psychological distress scores between PWH and their family members. Additionally, Pearson’s correlation was utilized to evaluate the bivariate associations between PWH’s and family members’ internalized stigma, resilience, and psychological distress.

To examine the dyadic mediation model among internalized stigma, resilience, and psychological distress between PWH and their family members, the distinguishable Actor-Partner Interdependence Mediation Model (APIMeM) was conducted. Two independent variables (PWH’s internalized stigma and family members’ courtesy internalized stigma), two dependent variables (PWH’s and family members’ psychological distress), and two possible mediator variables (PWH’s and family members’ resilience) compensated for the APIMeM’s two parts and its six variables. The intrapersonal (actor effects) and dyadic associations (partner effects) between these study variables were tested using standardized path coefficients derived from the APIMeM. The model was run using maximum likelihood. A 5000-sample, bias-corrected bootstrapping approach with a 95% confidence interval was used to calculate the mediation effects (i.e., actor and partner indirect effects). In terms of the model’s goodness of fit, the comparative fit index (CFI), Tucker-Lewis index (TLI), standardized root mean square residual (SRMR), and root mean square error of approximation (RMSEA) were used for verification. The model was considered to have a good fit to the data when CFI and TLI were larger than 0.95, and SRMR and RMSEA were lower than 0.08 [43]. Covariates tested included (1) age group, sex at birth, ethnicity, rurality, education level, occupation, and income level of both PWH and their family members; (2) family members’ HIV status; and (3) PWH’s HIV transmission mode. Model comparisons were conducted to assess the impact of these covariates on standardized coefficients and overall model fit. All analyses were conducted using R version 4.4.1, and a *p*-value less than 0.05 was considered statistically significant in all tests. This study protocol was reviewed and approved by the University of South Carolina Institutional Review Board (Protocol# Pro00099388) and the Guangxi Institutional Review Board (Protocol#GXIRB2020-39-1).

## Results

### Sociodemographic Characteristics

As shown in Table 1, the mean age of PWH (*n* = 800) and family members (*n* = 800) was 55.20 (SD = 11.20) and 49.50 (SD = 13.10) years, respectively. Among family members, 676 (85.57%) were from immediate families (e.g., parents and spouses), and 114 (14.43%) were from the family of choice (e.g., friends). Among PWH, 58.00% of the participants were male, 90.24% lived in rural areas, and 73.25% reported acquiring HIV through sexual intercourse (i.e., HIV transmission mode: sex). Among family members, 48.87% were male, 87.34% lived in rural areas, 32.50% were infected with HIV, and over 80% had an educational attainment lower than high school (i.e., less than primary school, primary school, or middle school).

### PWH’s and Family Members’ Mean Scores of Internalized Stigma, Resilience, and Psychological Distress

Paired t-tests showed that the score of internalized stigma was significantly higher among PWH compared to their family members (15.70 [SD = 5.87] vs. 13.80 [SD = 5.48], *p* < 0.001). PWH’s average resilience score was 34.00 (SD = 8.67), which was significantly lower than that in the family members (36.10[SD = 8.48], *p* < 0.001). PWH’s average psychological distress was 5.24 [SD = 2.30], which was higher than the family members’ score of 5.02 [SD = 2.52], albeit the difference was not significant (*p* = 0.06). (Table 2)

### Correlations Among the Internalized Stigma, Resilience, and Psychological Distress of PWH and their Family Members

Table 3 presents the correlation matrix among the main study variables in the hypothesized APIMeM. The correlations between PWH’s and their family members’ internalized stigma (*r* = 0.18, *p* < 0.001), resilience (*r* = 0.19, *p* < 0.001), and psychological distress (*r* = 0.11, *p* < 0.01) were positive and statistically significant. PWH’s psychological distress was positively correlated with their own internalized stigma (*r* = 0.27, *p* < 0.001) and negatively correlated with resilience (*r* = −0.37, *p* < 0.001), but its correlations with their family members’ internalized stigma (*r* = 0.07, *p* = 0.06) and resilience (*r* = −0.04, *p* = 0.27) were not statistically significant. Additionally, family members’ psychological distress was positively and significantly correlated with both PWH’s internalized stigma (*r* = 0.10, *p* < 0.01) and their own courtesy internalized stigma (*r* = 0.45, *p* < 0.001).

### Dyadic Impact of Internalized Stigma on Psychological Distress via the Mediator Role of Resilience

**Fig. 2:**
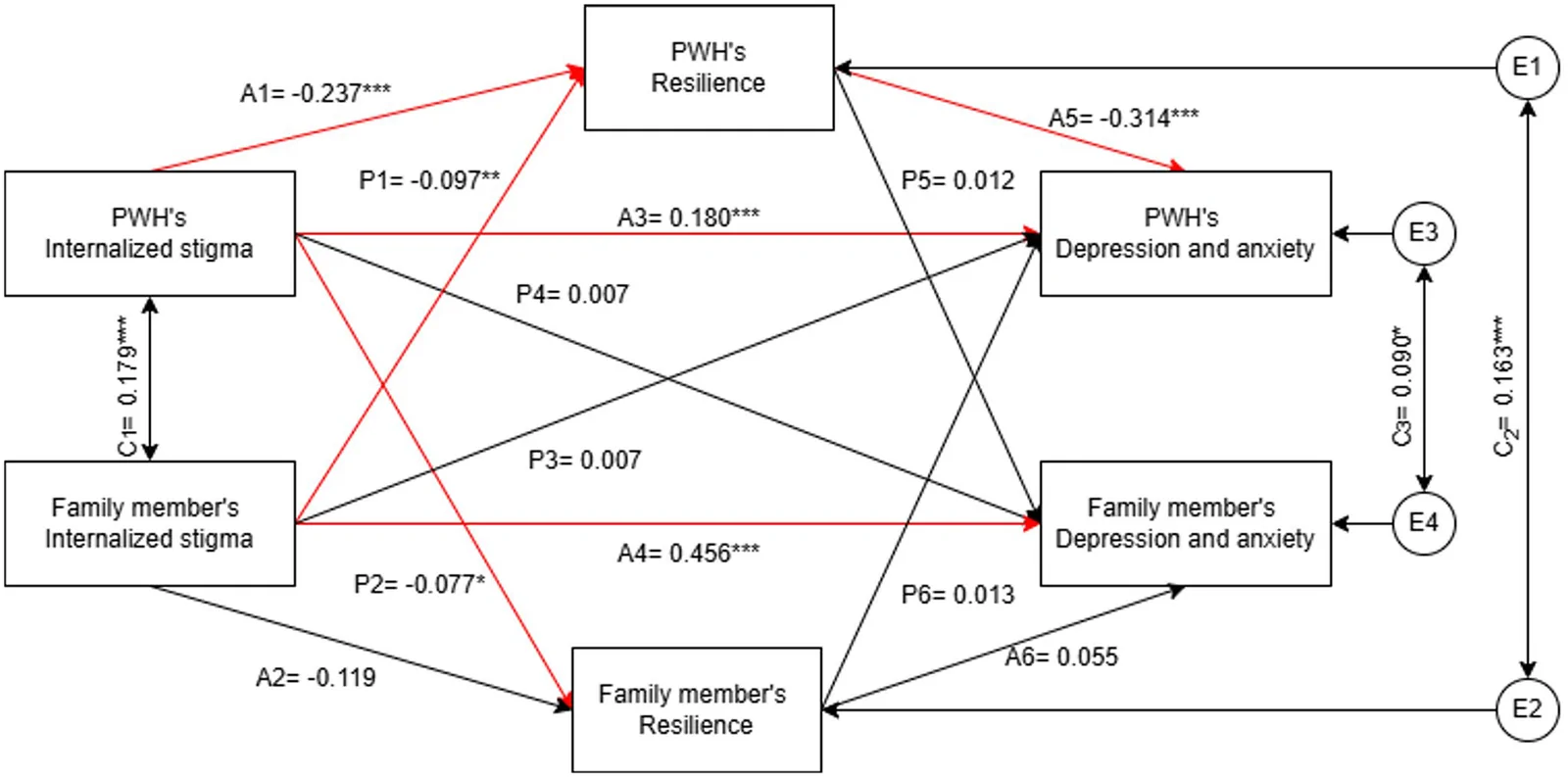
The actor-partner interdependence mediation model in people with HIV and their family members (*N* = 800 dyads)

Figure 2 shows standardized estimates for path coefficients and the significance of the APIMeM. The initial APIMeM was a saturated model, and then the partner effects of internalized stigma and resilience on psychological distress were constrained to be equal, given their similar standardized coefficients and levels of statistical significance in the unconstrained model. The Δχ^2^ test results indicated that the constrained model did not fit the data significantly worse than the unconstrained model [44]. We tested the following covariates in the model: (1) age group, sex at birth, ethnicity, rurality, education level, occupation, and income level of both PWH and their family members; (2) family members’ HIV status; and (3) PWH’s HIV transmission model. When covariates were controlled in the model, there was a slight change in the standardized coefficient; however, there was no significant change in the interpretation of the overall results, and the goodness of fit of the model was not improved. Therefore, the final model only included PWH’s and family members’ sex at birth as covariates after comprehensively considering the interpretation of results, the goodness of fit, and the principles of parsimony [45]. The final model showed an overall good fit to data: χ^2^ (27) = 19.35, *p* = 0.06; *TLI* = 0.96; *CFI* = 0.98; *SRMR* = 0.03; *RMSEA* = 0.03.

Regarding actor effects, the APIMeM suggested PWH’s internalized stigma was negatively associated with resilience (β = −0.24, SE = 0.05, *p* < 0.001) while positively associated with psychological distress (β = 0.18, SE = 0.01, *p* < 0.001). PWH’s resilience had a negative actor effect on psychological distress (β = −0.31, SE = 0.01, *p* < 0.001). The direct positive actor effect of Family members’ courtesy internalized stigma on psychological distress was significant (β = 0.46, SE = 0.06, *p* < 0.001). As for partner effects, the negative association between family members’ courtesy internalized stigma and PWH’s resilience (β = −0.10, SE = 0.05, *p* < 0.01) was significant. Additionally, higher PWH’s internalized stigma was significantly associated with lower family members’ resilience (β = −0.08, SE = 0.05, *p* = 0.04). (Fig. 2) Regarding the indirect actor effect, the indirect effect was significant between PWH’s internalized stigma and their psychological distress through their resilience (β = 0.08, SE = 0.01, *p* < 0.001). In terms of the indirect partner effect, the indirect effect was also significant between family members’ courtesy internalized stigma and PWH’s psychological distress through PWH’s resilience (β = 0.03, SE = 0.01, *p* = 0.01). (Table 4)

## Discussions

Guided by the Interdependence theory, the current study investigated the association between internalized stigma, resilience, and psychological distress of Chinese PWH and their family members from a dyadic perspective [23]. We found that resilience partially mediated the relationship between internalized stigma and psychological distress in PWH but not in their family members. Regarding partner effects, indirect effects were significant in the positive association between family members’ courtesy internalized stigma and PWH’s psychological distress through PWH’s resilience. By considering these dyadic associations within the family system, healthcare professionals in social services and communities need to emphasize a family-centered approach focusing on stigma reduction and resilience building among both PWH and their family members.

This study demonstrated that the positive direct actor effect of internalized stigma on psychological distress was significant for both PWH and family members. These findings highlight the deleterious impact of HIV internalized stigma, which not only impacts PWH but also extends to their close family members. Family members may experience HIV-related internalized stigma from their association with a PWH, and this type of courtesy stigma frequently manifests as a sense of identity loss, exclusion from community activities, and social rejection [46, 47]. Such experience may contribute to chronic psychological distress, including anxiety and depression. In addition, the dual burden of caring for PWH and managing stigma may place family members at an elevated risk for mental health challenges [48]. However, current stigma reduction strategies often overlook the needs of their family members and focus solely on PWH [48]. This gap underscores the need to develop family-centered interventions that reduce stigma and improve mental health not only for PWH but also for family members.

Our study suggested the indirect actor effect of internalized stigma leading to psychological distress through resilience was significant among PWH, which aligns with previous evidence [33, 49–51]. For example, a cross-sectional study on 402 PWH receiving care from a large immunology center in South Carolina showed that resilience significantly mediated the relationship between internalized HIV stigma and depression [29]. Individuals’ resilience level depends on multiple levels of resources, including individual (e.g., optimism and conscientiousness), interpersonal (e.g., social support), and community (e.g., community centers) levels [52, 53]. PWH may have limited access to these interpersonal or community resources and greater social isolation due to their fear of HIV disclosure, given the internalized stigma. These findings demonstrate that HIV-related stigma reduction and resilience promotion programs are needed for satisfactory mental health status among PWH.

Furthermore, this study found that family members’ courtesy internalized stigma had a significant indirect partner effect on PWH’s psychological distress through resilience. This finding suggests that courtesy stigma experienced by family members may indirectly contribute to poorer mental health outcomes for PWH by weakening resilience within the dyadic relationship. One possible explanation for this dynamic is that stigmatized PWH–family member dyads may experience diminished mutual support, relationship strain, and difficulty coping with external stressors [54]. For example, a study examining the actor and partner effects of microaggressions and internalized stigma on depression among same-sex male couples found that individuals with higher levels of internalized stigma were more likely to report negative relationship interactions, which in turn contributed to greater depressive symptoms in both themselves and their partners [55]. Similarly, internalized stigma within the PWH-family member dyad may foster feelings of withdrawal and emotional distance, further exacerbating psychological distress through weakened resilience. These challenges may be particularly pronounced in collectivist cultures, such as in rural Chinese culture, where family unity and social harmony are highly valued. In such a cultural context, HIV-related stigma can not only affect PWH but also extend to the family unit, leading to shared experiences of marginalization and limiting emotional support [56].

In this context, family-based interventions focusing on stigma reduction and resilience building are needed for PWH-family member dyads. Evidence from a systematic review of dyadic and family-oriented interventions for late-life depression suggests that dyadic approaches are often more effective than single-target interventions [57]. This is because dyadic interventions may enhance spousal functioning, such as supportive behaviors [58]. In Chinese culture, family relationships are often the primary source of support, further underscoring the potential benefits of dyadic interventions in this context [56]. Thus, family-centered stigma interventions would reduce psychological distress among PWH and their family members’ dyads by facilitating mutually supportive and reliable resilience.

The present study has some limitations. First, the cross-sectional design of this study restricts our ability to draw causal inferences or the directionality of associations among the variables. Psychological distress may influence one’s internalized stigma, rather than stigma leading to psychological distress. Utilizing follow-up data is suggested in future studies to explore these temporal and causal relationships. Second, the participants in this study were all affiliated with particular facilities and from only one province, which limits the generalizability of the findings to PWH in other geographic or cultural settings. Furthermore, the current study used baseline data from a resilience-based intervention study, which may be subject to participation bias. For example, PWH who agreed to enroll may perceive a stronger need to strengthen their resilience, family members who agreed to enroll may demonstrate a higher degree of support for their partner, and PWH who chose not to participate may be more likely to be socially isolated and unable to identify a willing family member. Thus, cautions are needed in generalizing the findings to all PWH and their family members.

Third, despite highly reliable measurement variables, all measures are self-reported, leading to potential recall bias or social desirability bias. Additionally, the measure of psychological distress would be limited by its brevity, as it only included 4 items and assessed two psychiatric symptoms (i.e., depression and anxiety). Future research would benefit from incorporating multiple measures of common symptoms in PWH (e.g., post-traumatic stress disorder) to provide a more comprehensive assessment of psychological distress. Fourth, this study focuses on internalized stigma, which may be shaped by broader structural and interpersonal experiences, such as perceived and enacted discrimination. Future research should explore these forms of stigma as well to better understand how they intersect and collectively influence psychological outcomes. Last, the findings of the dyadic model may primarily apply to immediate families since they comprised the majority of participants (84.5%). Our findings may not fully capture the potential unique dynamics of courtesy stigma, resilience, and psychological distress across different family relationships.

## Conclusions

This study expanded the literature by comprehensively examining the dyadic mechanism whereby internalized stigma of PWH and their family members affects mental health and by identifying various pathways. In summary, results showed that internalized stigma could impact psychological distress directly and indirectly, and resilience partly mediates this relationship among PWH. Furthermore, family members’ courtesy internalized stigma had an indirect partner effect on PWH’s psychological distress through PWH’s resilience. The mediating role of resilience and the observed partner effects suggest that interventions targeting courtesy stigma reduction among family members could have far-reaching benefits for PWH. Health professionals could adopt family-centered strategies that simultaneously focus on enhancing resilience and reducing stigma to effectively mitigate psychological distress and improve the overall well-being of both PWH and their family members.

## Figures and Tables

**Table 1:** Demographic and clinical characteristics of people with HIV-family member dyads

Characteristics	PWH (*n* = 800)	Family members(*n*=800)
	M ± SD or *n*(%)	M ± SD or *n*(%)
**Age (years)**	55.20 ± 11.20	49.50 (13.10)
**Age group**		
18–44	124 (15.50%)	309 (38.62%)
45–54	211 (26.38%)	184 (23.00%)
55–64	296 (37.00%)	194 (24.25%)
65+	169 (21.12%)	113 (14.13%)
**Sex**		
Male	464 (58.00%)	391 (48.87%)
Female	336 (42.00%)	409 (51.13%)
**Ethnicity**		
Han	496 (62.00%)	500 (62.50%)
Zhuang	267 (33.37%)	259 (32.37%)
Others^a^	367(4.63%)	40 (5.13%)
**Rural**		
Yes	721 (90.24%)	697 (87.34%)
No	78 (9.76%)	101 (12.66%)
**Education**		
Less than primary school	185 (23.13%)	104 (13.03%)
Primary school	285 (35.62%)	255 (31.95%)
Middle school	264 (33.00%)	283 (35.46%)
Higher school and higher	66 (8.25%)	156 (19.55%)
**Occupation**		
Employed	658 (82.25%)	668 (83.50%)
Other	36 (4.50%)	39 (4.87%)
Unemployed	106 (13.25%)	93 (11.63%)
**Income**		
Lower than average	424 (53.13%)	397 (49.69%)
Average	359 (44.99%)	381 (47.68%)
Above average	15 (1.88%)	21 (2.63%)
**HIV status**		
Positive		260 (32.50%)
Negative		491 (61.38%)
Unknown		49 (6.12%)
**Family type**		
Immediate family		676 (85.57%)
family of choice		114 (14.43%)
**HIV transmission**		
Sex	586 (73.25%)	
Blood donation	15 (1.88%)	
Injection drug use	17 (2.12%)	
Others/unknown	182 (22.75%)	

SD (standard deviation)

a Including Yao, Miao, Dai, and other ethnicities

**Table 2:** Internalized stigma, resilience, and psychiatric distress among people with HIV and family members (*N* = 800 dyads)

	Mean (Standard deviation)		Paired t (*p*-value)
	People with HIV	Family members	
Internalized stigma	15.70 (5.87)	13.80 (5.48)	8.32 (< 0.001)
Resilience	34.00 (8.67)	36.10 (8.48)	−5.86 (< 0.001)
Psychiatric distress	5.24 (2.30)	5.02 (2.52)	1.90 (0.058)

**Table 3:** Pearson correlations between internalized stigma, resilience, and psychiatric distress (*n* = 800)

	Internalized stigma (*r*(*p*))		Resilience [*r*(*p*)]		Psychiatric distress [*r*(*p*)]	
	*P*	F	*P*	F	*P*	F
P internalized stigma	1					
F internalized stigma	0.18 (*p* < 0.001)	1				
P resilience	−0.26 (*p* < 0.001)	0.14 (*p* < 0.001)	1			
F resilience	−0.09 (*p* = 0.01)	−0.13 (*p* < 0.001)	0.19 *(p* < 0.001)	1		
P psychiatric distress	0.27 (*p* < 0.001)	0.07 *(p* = 0.06)	−0.37 *(p* < 0.001)	−0.04 *(p* = 0.27)	1	
F psychiatric distress	0.10 (*p* = 0.01)	0.45 (*p* < 0.001)	−0.07 *(p* = 0.05)	0.00 *(p* = 0.98)	0.11 *(p* < 0.01)	1

P = People with HIV; F = Family members

**Table 4:** The direct, total, and indirect effects of the actor-partner interdependence mediation model (*N* = 800 dyads)

		B	β	SE	95%CI	*p*-value
**Direct effect**						
Actor effect	P internalized stigma -> P resilience	−0.344	−0.237	0.053	−0.446~−0.240	< 0.001
	P resilience -> P psychiatric distress	−0.085	−0.314	0.01	−0.105~−0.066	< 0.001
	P internalized stigma -> P psychiatric distress	0.07	0.18	0.014	0.043 ~ 0.098	< 0.001
	F internalized stigma -> F resilience	−0.184	−0.119	0.132	−0.454 ~ 0.060	0.163
	F resilience -> F psychiatric distress	0.016	0.055	0.031	−0.056 ~ 0.067	0.6
	F internalized stigma -> F psychiatric distress	0.21	0.456	0.056	0.078 ~ 0.299	< 0.001
Partner effect	P internalized stigma -> F resilience	−0.111	−0.077	0.053	−0.219~−0.008	0.038
	P resilience -> F psychiatric distress	0.004	0.012	0.006	−0.008 ~ 0.016	0.568
	P internalized stigma -> F psychiatric distress	0.003	0.007	0.009	−0.015 ~ 0.021	0.757
	F internalized stigma -> P resilience	−0.151	−0.097	0.052	−0.253~−0.051	0.003
	F resilience -> P psychiatric distress	0.004	0.013	0.006	−0.008 ~ 0.016	0.568
	F internalized stigma -> P psychiatric distress	0.003	0.007	0.009	−0.015 ~ 0.021	0.757
Total effect						
Actor effect	P internalized stigma -> P psychiatric distress	0.099	0.254	0.016	0.068 ~ 0.130	< 0.001
	F internalized stigma -> F psychiatric distress	0.207	0.449	0.051	0.107 ~ 0.307	< 0.001
Partner effect	F internalized stigma -> P psychiatric distress	0.015	0.036	0.01	−0.005 ~ 0.035	0.153
	P internalized stigma -> F psychiatric distress	0.001	0.003	0.01	−0.019 ~ 0.021	0.912
**Specific indirect effect**						
Actor effect	P internalized stigma -> P resilience -> P psychiatric distress	0.029	0.075	0.006	0.017 ~ 0.041	< 0.001
	F internalized stigma -> F resilience -> F psychiatric distress	−0.003	−0.007	0.007	−0.017 ~ 0.011	0.684
Partner effect	P internalized stigma -> P resilience -> F psychiatric distress	−0.001	−0.003	0.002	−0.005 ~ 0.003	0.576
	P internalized stigma -> F resilience -> P psychiatric distress	−0.000	−0.001	0.001	−0.002 ~ 0.002	0.614
	P internalized stigma -> F resilience -> F psychiatric distress	−0.002	−0.004	0.004	−0.010 ~ 0.006	0.642
	F internalized stigma -> F resilience -> P psychiatric distress	−0.001	−0.002	0.002	−0.005 ~ 0.003	0.71
	F internalized stigma -> P resilience -> F psychiatric distress	−0.001	−0.001	0.001	−0.003 ~ 0.001	0.605
	F internalized stigma -> P resilience -> P psychiatric distress	0.013	0.03	0.005	0.003 ~ 0.023	0.006

B, unstandardized regression coefficients; β, standardized coefficients

P, people with HIV; F, family members; SE, standard error; CI, confidence interval

## Data Availability

The data used in this study are not publicly available due to the inclusion of sensitive and confidential information related to patient data. Access to data may be granted by Dr. Xiaoming Li (email: xiaoming@mailbox.sc.edu) upon reasonable request and with appropriate institutional approvals.

## References

[1] Leserman J, Petitto JM, Gu H, Gaynes BN, Barroso J, Golden R, Progression to AIDS, a clinical AIDS condition and mortality: psychosocial and physiological predictors. Psychol Med. 2002;32(6):1059–73. 10.1017/s0033291702005949 12214787

[2] Sun W, Wu M, Qu P, Lu C, Wang L. Psychological well-being of people living with HIV/AIDS under the new epidemic characteristics in China and the risk factors: a population-based study. Int J Infect Dis. 2014;28:147–52. 10.1016/j.ijid.2014.07.010 25281441

[3] Ma H, Zhu F, Zhai H, Ma Y, Liu Y, Wang S, Xu Y. Prevalence of psychological distress among people living with HIV/AIDS: a systematic review and meta-analysis. AIDS Care. 2023;35(2):153–64. 10.1080/09540121.2022.2080802 35642250

[4] Hu F-H, Liu P, Jia Y-J, Ge M-W, Shen L-T, Xia X-P, Prevalence of mental health problems in people living with HIV: a systematic review and meta-analysis. Psychol Health Med. 2024. 10.1080/13548506.2024.2424998. 39504439

[5] Gooden TE, Gardner M, Wang J, Chandan JS, Beane A, Haniffa R, The risk of mental illness in people living with HIV in the UK: a propensity score-matched cohort study. Lancet HIV. 2022;9(3):e172–81. 10.1016/S2352-3018(21)00319-2 35123667

[6] Olagunju AT, Ogundipe OA, Erinfolami AR, Akinbode AA, Adeyemi JD. Toward the integration of comprehensive mental health services in HIV care: an assessment of psychiatric morbidity among HIV-positive individuals in sub-Saharan Africa. AIDS Care. 2013;25(9):1193–8. 10.1080/09540121.2013.763892 23391152

[7] Dipio R, Acuda W, Namisango E, Nalubega-Mbowa MG. Prevalence and factors associated with depressive symptoms among family caregivers of palliative care patients at Hospice Africa Uganda. Palliat Support Care. 2022;20(3):375–82. 10.1017/S1478951521000730 34154696

[8] Pequegnat W, Bray JH. HIV/STD prevention interventions for couples and families: a review and introduction to the Special Issue. Couple Fam Psychol Res Pract. 2012;1(2):79.

[9] Hao C, Liu H. Actor and partner effects of perceived HIV stigma on social network components among people living with HIV/AIDS and their caregivers. Glob Health Promot. 2015;22(2):40–52. 10.1177/1757975914537321 PMC4312745 25085478

[10] Zhang Y, Liu M, Han J, Tian X, Xin Y. Beyond the burden: a qualitative inquiry into the experiences of Chinese informal caregivers for people living with HIV or AIDS. Patient Prefer Adherence. 2024;677:85. 10.2147/PPA.S454590 PMC10949991 38505188

[11] Ji G, Li L, Lin C, Sun S. The impact of HIV/AIDS on families and children-a study in China. AIDS. 2007;21:S157–61. 10.1097/01.aids.0000304712.87164.42 PMC2822872 18172385

[12] Li L, Liang L-J, Ding YY, Ji G. Facing HIV as a family: predicting depressive symptoms with correlated responses. J Fam Psychol. 2011;25(2):202. 10.1037/a0022755 PMC3076685 21480700

[13] Thapinta D, Srithanaviboonchai K, Uthis P, Suktrakul S, Wiwat-wongnawa R, Tangmunkongvorakul A, Association between internalized stigma and depression among people living with HIV in Thailand. Int J Environ Res Public Health. 2022;19(8):4471. 10.3390/ijerph19084471 PMC9031422 35457339

[14] O’Donnell AT, Foran A-M. The link between anticipated and internalized stigma and depression: a systematic review. Soc Sci Med. 2024. 10.1016/j.socscimed.2024.116869. 38678910

[15] Rueda S, Mitra S, Chen S, Gogolishvili D, Globerman J, Chambers L, Examining the associations between HIV-related stigma and health outcomes in people living with HIV/AIDS: a series of meta-analyses. BMJ Open. 2016;6(7):e011453. 10.1136/bmjopen-2016-011453 PMC4947735 27412106

[16] Van der Kooij YL, den Daas C, Bos AE, Willems RA, Stutterheim SE. Correlates of internalized HIV stigma: a comprehensive systematic review. AIDS Educ Prev. 2023;35(2):158–72. 10.1521/aeap.2023.35.2.158 37129595

[17] Khan R, Pai K, Kulkarni V, Ramapuram J. Depression, anxiety, stress and stigma in informal caregivers of people living with HIV (PLHIV). AIDS Care. 2018;30(6):722–6. 10.1080/09540121.2017.1418831 29278924

[18] Algarin AB, Sheehan DM, Varas-Diaz N, Fennie K, Zhou Z, Spencer EC, Enacted HIV-related stigma’s association with anxiety & depression among people living with HIV (PLWH) in Florida. AIDS Behav. 2021;25(1):93–103. 10.1007/s10461-020-02948-5 PMC7749818 32564164

[19] Goffman E Stigma: Notes on the management of spoiled identity. Simon and schuster; 2009.

[20] Liu H, Xu Y, Sun Y, Dumenci L. Measuring HIV stigma at the family level: Psychometric assessment of the Chinese Courtesy Stigma Scales (CCSSs). PLoS One. 2014;9(3):e92855. 10.1371/journal.pone.0092855 PMC3962465 24658364

[21] Williams LD, Aber JL, Group SR. The multilevel relationships of HIV-related stigma to child and caregiver mental health among HIV-affected households in South Africa. Am J Community Psychol. 2019;63(1–2):3–16. 10.1002/ajcp.12280 30368830

[22] McHenry MS, Nyandiko WM, Scanlon ML, Fischer LJ, McAteer CI, Aluoch J, HIV stigma: perspectives from Kenyan child caregivers and adolescents living with HIV. J Int Association Providers AIDS Care (JIAPAC). 2017;16(3):215–25. 10.1177/2325957416668995 PMC5464367 27655835

[23] Bagheri Z, Taheri M, Motazedian N. The impacts of depression and anxiety on quality of life among patients with HIV/AIDS and their spouses: testing dyadic dynamics using the actor-partner interdependence model. AIDS Care. 2019. 10.1080/09540121.2019.1595676. 30884955

[24] Yu NX, Chan CL, Zhang J. Dyadic effects of stigma and discrimination on distress in Chinese HIV discordant couples. AIDS Educ Prev. 2016;28(4):277–86. 10.1521/aeap.2016.28.4.277 27427923

[25] Liu H, Xu Y, Lin X, Shi J, Chen S. Associations between perceived HIV stigma and quality of life at the dyadic lvel: the actor-partner interdependence model. PLoS One. 2013;8(2):e55680. 10.1371/journal.pone.0055680 PMC3562178 23383343

[26] Huang J, Zhang J, Yu NX. Couple identity and well-being in Chinese HIV serodiscordant couples: resilience under the risk of stigma. AIDS Care. 2018;30(sup5):S58–66. 10.1080/09540121.2018.1510105 30632776

[27] Kawi J, Reyes AT, Arenas RA. Exploring pain management among Asian immigrants with chronic pain: Self-management and resilience. J Immigr Minor Health. 2019;21:1123–36. 10.1007/s10903-018-0820-8 30182206

[28] Waugh CE, Koster EH. A resilience framework for promoting stable remission from depression. Clin Psychol Rev. 2015;41:49–60. 10.1016/j.cpr.2014.05.004 24930712

[29] Brown MJ, Gao C, Kaur A, Qiao S, Li X. Social support, internalized HIV stigma, resilience and depression among people living with HIV: a moderated mediation analysis. AIDS Behav. 2023;27(4):1106–15. 10.1007/s10461-022-03847-7 PMC10115436 36094638

[30] Yan H, Li X, Li J, Wang W, Yang Y, Yao X, Association between perceived HIV stigma, social support, resilience, self-esteem, and depressive symptoms among HIV-positive men who have sex with men (MSM) in Nanjing, China. AIDS Care. 2019;31(9):1069–76. 10.1080/09540121.2019.1601677 30942086

[31] Li X, Yan H, Wang W, Yang H, Li S. Association between enacted stigma, internalized stigma, resilience, and depressive symptoms among young men who have sex with men in China: a moderated mediation model analysis. Ann Epidemiol. 2021;56:1–8. 10.1016/j.annepidem.2021.01.001 33422600

[32] Yuan GF, Zhang R, Qiao S, Li X, Zhou Y, Shen Z. Longitudinal analysis of the relationship between internalized HIV stigma, perceived social support, resilience, and depressive symptoms among people living with HIV in China: a four-wave model. AIDS Behav. 2024;28(2):645–56. 10.1007/s10461-023-04251-5 38091128

[33] Zhang L, Li X, Qiao S, Zhou Y, Shen Z, Tang Z, The mediating role of individual resilience resources in stigma–health relationship among people living with HIV in Guangxi, China. AIDS Care 2015;27(10):1317–25. 10.1080/09540121.2015.1054338 PMC6234005 26274908

[34] Ke J, Lin J, Lin X, Chen W-t, Huang F. Dyadic effects of family resilience on quality of life in patients with lung cancer and spousal caregivers: the mediating role of dyadic coping. Eur J Oncol Nurs. 2023;66:102400. 10.1016/j.ejon.2023.102400 37611499

[35] Qin F, Wei T, Zhao X, He Y, Chen M, Luo Z, Relationship between family resilience and dyadic coping in colorectal cancer patients and their spouses, based on the actor-partner interdependence model. Eur J Oncol Nurs. 2024;70:102622. 10.1016/j.ejon.2024.102622 38795443

[36] Gamarel KE, Revenson TA. Dyadic adaptation to chronic illness: The importance of considering context in understanding couples’ resilience. In: Couple resilience: Emerging perspectives. Springer; 2015. p. 83–105.

[37] Li X, Qiao S, Yang X, Harrison SE, Tam CC, Shen Z, A resilience-based intervention to mitigate the effect of HIV-related stigma: protocol for a stepped wedge cluster randomized trial. Front Public Health. 2022;10:857635. 10.3389/fpubh.2022.857635 PMC9001957 35425746

[38] Yuan GF, Tam CC, Yang X, Qiao S, Li X, Shen Z, Zhou Y. Associations between internalized and anticipated HIV stigma and depression symptoms among people living with HIV in China: a four-wave longitudinal model. AIDS Behav. 2023;27(12):4052–61. 10.1007/s10461-023-04119-8 37392272

[39] Berger BE, Ferrans CE, Lashley FR. Measuring stigma in people with HIV: Psychometric assessment of the HIV stigma scale. Res Nurs Health. 2001;24(6):518–29. 10.1002/nur.10011 11746080

[40] Campbell-Sills L, Stein MB. Psychometric analysis and refinement of the connor–davidson resilience scale (CD-RISC): validation of a 10-item measure of resilience. J Trauma Stress: Official Publication Int Soc Trauma Stress Stud. 2007;20(6):1019–28. 10.1002/jts.20271 18157881

[41] Wang L, Shi Z, Zhang Y, Zhang Z. Psychometric properties of the 10-item Connor–Davidson Resilience Scale in Chinese earthquake victims. J Neuropsychiatry Clin Neurosci. 2010;64(5):499–504. 10.1111/j.1440-1819.2010.02130.x 20923429

[42] Kroenke K, Spitzer RL, Williams JB, Löwe B. An ultra-brief screening scale for anxiety and depression: the PHQ–4. Psychosomatics. 2009;50(6):613–21. 10.1176/appi.psy.50.6.613 19996233

[43] Barrett P Structural equation modelling: adjudging model fit. Pers Indiv Differ. 2007;42(5):815–24.

[44] Kenny DA, Ledermann T. Detecting, measuring, and testing dyadic patterns in the actor–partner interdependence model. J Fam Psychol. 2010;24(3):359. 10.1037/a0019651 20545409

[45] Kim Y, Chae H. Associations among family strengths, depression and life satisfaction between disabled children and their parent caregivers: an actor–partner interdependence mediation model. J Adv Nurs. 2024. 10.1111/jan.16474. 39306832

[46] Maldonado DAC, Martins LF, Ronzani TM. Courtesy stigma and health conditions: systematic literature review. Psicologia em Estudo. 2023;28:e52111.

[47] Robert LM. HIV and AIDS-related courtesy stigma: South African caregivers’ experiences and coping strategies. Alternation J. 2016;23(2):120–40.

[48] Ma PH, Chan ZC, Loke AY. Self-stigma reduction interventions for people living with HIV/AIDS and their families: a systematic review. AIDS Behav. 2019;23:707–41. 10.1007/s10461-018-2304-1 30298241

[49] Domlyn AM, Jiang Y, Harrison S, Qiao S, Li X. Stigma and psychosocial wellbeing among children affected by parental HIV in China. AIDS Care. 2020;32(4):500–7. 10.1080/09540121.2019.1687834 31690083

[50] Chi P, Li X, Du H, Tam CC, Zhao J, Zhao G. Does stigmatization wear down resilience? A longitudinal study among children affected by parental HIV. Pers Indiv Differ. 2016;96:159–63.

[51] Balaji AB, Bowles KE, Hess KL, Smith JC, Paz-Bailey G. Association between enacted stigma and HIV-related risk behavior among MSM, National HIV behavioral surveillance system, 2011. AIDS Behav. 2017;21(1):227–37. 10.1007/s10461-016-1599-z 27830344

[52] Dulin AJ, Dale SK, Earnshaw VA, Fava JL, Mugavero MJ, Napravnik S, Resilience and HIV: a review of the definition and study of resilience. AIDS Care. 2018;30(sup5):S6–17. 10.1080/09540121.2018.1515470 PMC6436992 30632778

[53] Woodward EN, Banks RJ, Marks AK, Pantalone DW. Identifying resilience resources for HIV prevention among sexual minority men: a systematic review. AIDS Behav. 2017;21(10):2860–73. 10.1007/s10461-016-1608-2 27981398

[54] Sarno EL, Bundy C, Dyar C, Newcomb ME. Examining minority stress, dyadic coping, and internalizing symptoms among male same-sex couples using actor–partner interdependence models. J Couns Psychol. 2021;68(5):515. 10.1037/cou0000542 PMC8455724 33749295

[55] Feinstein BA, McConnell E, Dyar C, Mustanski B, Newcomb ME. The influence of stress on depression and substance use problems among young male same-sex couples: relationship functioning as an underlying mechanism. Clin Psychol Sci. 2019;7(5):928–40. 10.1177/2167702619842561 PMC6774625 31579559

[56] Xu A, Xie X, Liu W, Xia Y, Liu D. Chinese family strengths and resiliency. Marr Fam Rev. 2007;41(1–2):143–64.

[57] Stahl ST, Rodakowski J, Saghafi EM, Park M, Reynolds CF, Dew MA. Systematic review of dyadic and family-oriented interventions for late-life depression. Int J Geriatr Psychiatry. 2016;31(9):963–73. 10.1002/gps.4434 PMC5166608 26799782

[58] Martire LM, Schulz R, Helgeson VS, Small BJ, Saghafi EM. Review and meta-analysis of couple-oriented interventions for chronic illness. Ann Behav Med. 2010;40(3):325–42. 10.1007/s12160-010-9216-2 PMC4101802 20697859

